# Establishment of an Immortalized Canine Hippocampal Neural Stem Cell Line via SV40LT Retroviral Transduction

**DOI:** 10.3390/cells15060543

**Published:** 2026-03-19

**Authors:** Yankun Ke, Zixin Li, Huaiyu Wang, Yixuan Zhang, Shiyu Xu, Longlong Zhang

**Affiliations:** 1 Institute of Biomedical Engineering, Kunming Medical University, Kunming 650500, China; 2 Faculty of First Clinical Medical, Kunming Medical University, Kunming 650500, China; 3 Kunming Institute of Zoology, Chinese Academy of Sciences, Kunming 650201, China

**Keywords:** NSCs, dog, hippocampus, SV40LT, immortalization

## Abstract

Dogs represent a promising animal model for analyzing human neurodegenerative diseases, owing to their similarities to humans in nervous system architecture and behavioral phenotypes. Neural stem cells (NSCs) serve as a highly valuable in vitro experimental model for investigating neurogenesis, neurodegenerative disease pathogenesis, and neural molecular biology; however, studies on immortalized canine neural stem cell lines remain scarce. Herein, we successfully established an immortalized canine hippocampal neural stem cell line that can be continuously passaged in vitro via SV40 large T antigen (SV40LT) viral infection and subsequent cellular transformation. Both the immortalized NSCs and their normal parental counterparts differentiated into neuronal and glial lineages under induced differentiation conditions. Normal canine hippocampal NSCs can be passaged for no more than 10 generations, whereas the immortalized line can be passaged indefinitely while maintaining a normal karyotype. This immortalized canine hippocampal NSC line can act as a critical experimental tool for future research into neural differentiation mechanisms and stem cell-derived therapeutic strategies for neurological disorders in dogs.

## 1. Introduction

Neural stem cells (NSCs) are characterized by their multipotency and capacity for self-renewal and they undergo differentiation into neurons, astrocytes, and oligodendrocytes under certain conditions in the nervous system [1]. Given their critical roles in the nervous system’s development, maintenance, and repair [2], they are promising tools for functional studies, drug discovery, and cell transplantation therapy [3]. In the mammalian nervous system, NSCs are present during both development and adulthood [4], generating functional neurons or glial cells in the subventricular zone (SVZ) of the lateral ventricle and in the subgranular zone (SGZ) of the dentate gyrus in the hippocampus. NSCs generate functional neurons or glial cells throughout the lives of most mammals [5,6]. Adult-born neurons from the hippocampus NSCs have been widely used to study neuronal development; dysfunctions in human hippocampus neurogenesis have been associated with several human neurological and psychiatric diseases [7]. This fact, in addition to the hippocampus’ distinct structure and specialized functions, has attracted a great deal of attention from researchers worldwide.

NSCs have been extensively isolated and propagated in vitro from the embryonic, neonatal, and adult animal central nervous systems [8]. However, primary NSCs cannot be expanded indefinitely in this environment [9]. For primary cultures, the limited proliferative lifespan of primary cells, a phenomenon known as the ‘Hayflick limit’, is a major restriction [10]. Telomerase activity maintains chromosomal telomere length, thereby enabling cells to retain mitotic competence. As telomerase levels decline, telomeres shorten, progressively limiting and ultimately preventing mitosis [11]. Several kinds of immortalized NSCs (iNSCs) have been established with high proliferative potential while maintaining neural differentiation capacity [8,12,13,14,15]; they retain stable stem cell characteristics and are no longer limited by proliferation. Sourcing iNSCs from a variety of species therefore significantly improves their utility for basic research and stem cell therapy.

In the present study, we extracted a canine neural stem cell line (CNSC) from the hippocampal cells of three 5-month-old Beagles, and subsequently infected the primary cells with a retrovirus expressing simian virus 40 large T (SV40LT) antigen to construct an immortalized canine neural stem cell (CNSC-T) line. We found that the morphology and surface makers of the CNSC-Ts were the same as that of the primary CNSCs and that CNSC-Ts possessed a similar differentiation ability; however, they had a greater capacity for proliferation than the CNSCs. Thus, our cell line provides an efficient model to elucidate the regulatory roles of intrinsic and extrinsic factors in NSC biology and their impact on neuronal diversification, making it a valuable resource for both basic research and the discovery of novel drug targets for neurodegenerative diseases.

## 2. Materials and Methods

### 2.1. Isolation and Culture of NSCs and Cell Proliferation Assay

Three 5-month-old female Beagles were obtained from Kunming Medical University [16]. The hippocampal tissues were isolated by Qijun Zhou and the animal procedures were approved by the Animal Ethics Committee of Kunming Institute of Zoology, Chinese Academy of Sciences (KIZ, CAS) (Approval code: SMKX-20 160 301-01; Approval date: 1 March 2016). Primary CNSCs were isolated from bilateral hippocampal tissues of three 5-month-old female Beagles according to our previous study [17]. The entire hippocampal tissue was cut into pieces, subsequently digested with Accutase (Life Technologies), and then cultured in non-coated 60 mm dishes with the proliferative medium (50% Neural Basal, 50% DMEM-F12/Glutamax, 1× N2, 1× B27, 0.1 mmol/L Non-Essential Amino Acids, 20 ng/mL EGF, 20 ng/mL bFGF, and 100× penicillin/streptomycin). Neurospheres formed after 7 days (passage 0) and were subsequently passaged three times with Accutase to eliminate contaminating cells. For adherent culture, the CNSCs were seeded onto 20 μg/mL poly-L-ornithine- (Thermo Fisher Scientific) and 10 μg/mL laminin- (STEMCELL^TM^ Technologies) coated dishes. Proliferation was assessed by counting cells plated in 6-well plates at regular intervals. For cryopreservation, cells were frozen in a medium consisting of 90% culture medium and 10% DMSO. Thawing was performed by rapidly warming the frozen cells in a 37 °C water bath.

### 2.2. Immunofluorescent (IF) and Immunohistochemistry (IHC) Staining

Cells cultured in coated chambers were fixed with 4% paraformaldehyde for 30 min, then washed at least three times with PBS (phosphate-buffered saline). After permeabilization with 0.1% Triton X-100 for 20 min and blocking with 10% normal goat serum for 1 h, the cells were incubated with primary antibodies overnight at 4 °C. The next day, the cells were incubated with DyLight 488- or DyLight 555-labeled secondary antibodies (1:400, Thermo Fisher) and 4′,6-diamidino-2-phenylindole (DAPI) for 1–2 h at room temperature. Stained cells were visualized and imaged using a laser-scanning confocal microscope (Olympus, Tokyo, Japan). The numbers of Ki67^+^ cells were quantified with Image J software.

Formalin-fixed and paraffin-embedded hippocampal tissue specimens were sliced into 5–7 μm sections, deparaffinized in turpentine oil (TO), and rehydrated through a graded ethanol series. Antigen retrieval was then performed by heating the slides at 95 °C for 40 min in the 10 mmol/L sodium citrate solution (Thermo Fisher), followed by cooling to room temperature. For each hippocampal tissue, at least five sections encompassing the hippocampal region were analyzed, with 5–6 optical fields examined per section. The slides were washed with phosphate-buffered saline (PBS) and permeabilized with 1% Triton X-100 solution for 10 min. Slides were blocked with 10% BSA for 1–2 h at room temperature and incubated with primary antibodies overnight at 4 °C. For immunofluorescence staining, slides were incubated for 2 h at room temperature with DAPI and DyLight 488- or DyLight 555-conjugated secondary antibodies (1:400, Thermo Fisher Scientific, Waltham, MA, USA). For immunohistochemistry, slides were incubated with HRP-conjugated secondary antibodies for 0.5 h, followed by the addition of 3,3′-diaminobenzidine (DAB) substrate until the desired staining intensity was achieved. The sections were then lightly counterstained with hematoxylin. Finally, all slides were dehydrated through a graded ethanol series and mounted with coverslips. Stained slides were visualized and imaged using a laser-scanning confocal microscope (LSCM, Olympus Corporation, Tokyo, Japan).

The following primary antibodies were used in this study: rabbit anti-Ki67 (Abcam, Cambridge, MA, USA, Cat# ab15580), rabbit glial fibrillary acidic protein (GFAP) (Dako, Cat# Z0334), rabbit platelet-derived growth factor receptor alpha (PDGFRα) (Sigma, Cat# SAB4502142), rabbit calcyphosine 2 (CADPS2) (Millipore, Cat# ABN326), mouse anti-Nestin (Abcam, Cat# ab6142), rabbit anti-β-tubulin III (Biolegend, San Diego, CA, USA, Cat# PRB-435P), mouse anti-MAP2 (Sigma-Aldrich, St. Louis, MO, USA, Cat# M9942), goat anti-Sox2 (Santa Cruz Biotechnology, Dallas, TX, USA, Cat# sc-17320), mouse anti-O4 (R&D, Cat# MAB1326), rabbit anti-Synapsin-1 (Millipore, Cat# AB1543), rabbit anti-SV40 T Antigen (MedChemExpress, Cat# HY-P83511), mouse anti-Vimentin (Thermo Fisher, Cat# OMA1-06001), and mouse anti-α-tubulin (ProteinTech, Rosemont, IL, USA, Cat# 11224-1-AP).

### 2.3. Neural Stem Cell Differentiation

NSCs were seeded at 6000–8000 cells per well onto poly-L-ornithine and laminin-coated chambers in proliferation medium. After 12 h, the medium was switched to differentiation medium (50% Neural Basal, 50% DMEM-F12/Glutamax, 1× N2, 1× B27, 0.075% BSA, 0.1 mmol/L Non-Essential Amino Acids, 200 mol/L ascorbic acid, 2 mol/L db-cAMP, 20 ng/mL BDNF, 20 ng/mL GDNF, and 100× penicillin/streptomycin) [18]. The medium was refreshed every other day. Immunofluorescence staining assay was performed after differentiation.

### 2.4. Karyotype Analysis

Karyotype analysis was conducted by Wenhui Nie at the Cell Bank of the Kunming Institute of Zoology, Chinese Academy of Sciences. Briefly, 0.1 mL of Colcemid (Sigma) is added to the culture medium to induce metaphase arrest in CNSC-Ts at 37 °C for 2–4 h. Cells are then harvested using trypsin and incubated with 75 mM KCl hypotonic solution at 37 °C for 30 min. Ice-cold fixative (methanol:acetic acid, 3:1) is added for repeated fixation three times. The cell suspension is dropped onto slides from a height of 30–50 cm to obtain metaphase spreads. After Giemsa (Sigma) staining for 15 min, the slides are air-dried and examined under a microscope. Chromosomes are then digitally arranged into a karyogram.

### 2.5. FACS and Cell Cycle Analysis

Collected NSCs were rinsed twice with PBS and fixed in ice-cold 70–80% ethanol for 72 h. Following two further washes with ice-cold PBS, cells were incubated in PBS containing 50 μg/mL propidium iodide (Sigma) and 20 μg/mL RNase A at 37 °C for 0.5 h. The stained cells were examined with a BD LSR Fortessa flow cytometer (San Jose, CA, USA), and the acquired data were analyzed with FlowJo VX software.

### 2.6. Western Blot Analysis

The cells were lysed in RIPA (CWBIO, China) lysis buffer, and the total proteins were fractionated by 12% SDS-PAGE (polyacrylamide gel electrophoresis). The membranes were blocked with 5% BSA. The membranes were incubated with the primary antibodies and subsequently with secondary antibodies.

### 2.7. Retroviral Infection and RT-PCR

SV40LT retroviral plasmids was provided from Ms. Chunli Sun at Kunming Institute of Zoology, Chinese Academy of Sciences, China. In detail, retroviruses were produced by co-transfecting plasmid pBABE-puro SV40LT together with packaging and envelope plasmids (pCL-Eco, pCL-Ampho and pCMV-VSV-G), with a total of 25 μg of DNA, into HEK293T cells using polyethyleneimine (PEI). The culture medium was changed 8 h after transfection. Supernatant containing SV40LT virus was collected at 48 and 72 h after the medium change, respectively. Approximately 5 × 10^6^ of low-passage CNSCs in adherent culture were used for viral infection. Cells were infected once or twice with SV40LT retroviruses containing 4 μg/mL polybrene, which increases infection efficiency. Cells infected with retroviruses were selected by 1 μg/mL of puromycin (Solarbio, China) to obtain stably infected cell lines. The methods of RT-PCR were used as previously described [19]. The PCR primers were used as follows: SV40LT-Forwad, 5′-TGTGGTATGGCTGATTATGA-3′; SV40LT-Reverse, 5′-CGCAGTGAGTTTTTG TTAGA-3′. The length of amplified fragment is 372 bp and the annealing temperature is 55 °C.

## 3. Results

### 3.1. Staining of Different Neural Cells in Canine Hippocampal Tissue

**Figure 1:**
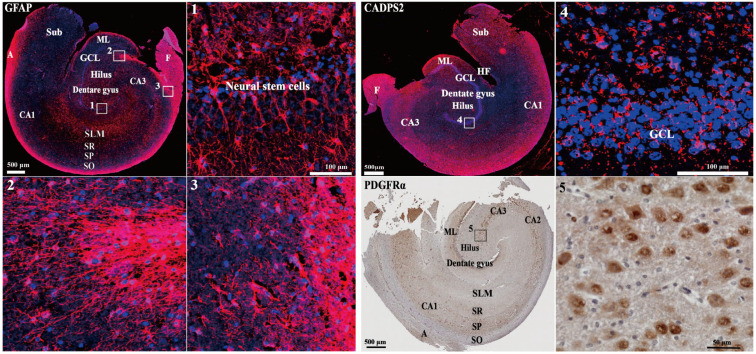
The expressions of GFAP, CADPS2, and PDGFRα in the dog hippocampus revealed by immunofluorescent and immunohistochemical staining. Red and brown points indicate cells in which the proteins were expressed. Red: GFAP and CADPS2; blue: DAPI; brown: PDGFα. DG, dentate gyrus; GCL, granule cell layer, SGZ, subgranular zone; ML, molecular layer; CA, cornu ammonis; SO, stratum oriens; SP, stratum pyramidale; SR, stratum radiatum; SLM, stratum lacunosum-moleculare; Sub, subiculum; HF, hippocampus fissure; A, alveus; F, Fimbria.

In the adult hippocampus, neural stem cells (NSCs) in the dentate gyrus (DG) exist primarily as radial glia-like cells (RGLs). These cells represent the major population of quiescent NSCs and are distinctly characterized by the co-expression of GFAP, Nestin and Sox2 [20,21]. We first confirmed the existence of neural stem cells in the DG zone using IF staining. GFAP fluorescence intensity remained stable in the F (Fimbria), A (alveus), DG (dentate gyrus), CA (cornu ammonis) and SLM (stratum lacunosum-moleculare) regions of the hippocampus (Figure 1). Typically, neural stem cells derived from the subgranular zone (SGZ) also exhibited a GFAP-positive morphology, yet they were significantly distinct from GFAP-positive astrocytes in other regions (Figure 1). The expression patterns of GFAP-positive cells identified in the canine hippocampal tissue were similar to that observed in the adult mice [22]. These results demonstrated that a large number of primary NSCs exist in the hippocampus of a 5-month-old Beagle.

Additionally, we also examined the expression patterns of neurons and oligodendrocytes. Calcium-dependent secretion activator 2 (CADPS2), a molecular marker that specifically labels neuropeptide/neurotrophic factor-secreting neurons, plays an important role in secretory granule exocytosis. It is a molecular marker that specifically labels neuropeptide/neurotrophic factor-secreting neurons, and its expression pattern is highly coupled with the secretory function of dense-core vesicles (DCVs) [23]. This marker holds significant application value for the study of neural development, synaptic plasticity and neuropsychiatric disorders. As shown in Figure 1, CADPS2 is widely and highly expressed throughout the canine hippocampal tissue, particularly in the CA regions, except for a localized region adjacent to the hippocampal fissure (HF) where expression is relatively weaker. In mouse hippocampi, CADPS2 is enriched at high levels in the CA3 and DG regions [24], showing reduced distribution and function compared to the hippocampi of dogs.

Oligodendrocyte progenitor cells (OPCs) play a crucial role in maintaining the neogenesis of oligodendrocytes and the formation of myelin sheaths. PDGFRα, an OPC marker gene, was highly expressed in our immunohistochemical results, particularly in the GCL (granule cell layer), ML (molecular layer), and all CA region layers in the canine hippocampus (Figure 1). In comparison, PDGFRα expression in the mouse hippocampus was relatively even in the CA1 and CA3 subregions and the dentate gyrus, and it was widely expressed across all layers of each hippocampal CA subregion [25].

### 3.2. Isolation and Screening of Canine Hippocampal Neural Stem Cells

**Figure 2:**
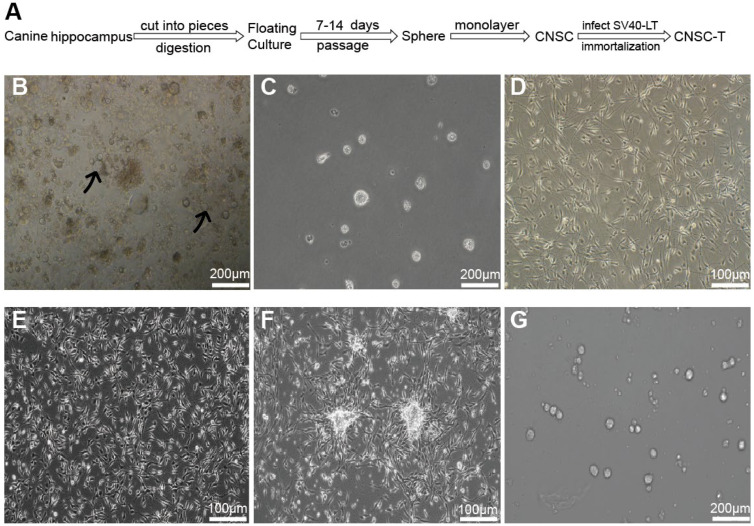
The morphological characteristics of CNSCs and CNSC-Ts. (**A**) Schematic diagram of isolation and culture of neural stem cells from canine hippocampal tissue. (**B**) The first generation of neurospheres with floating culture, with the black arrows showing the neurospheres that grow in the space among tissue scraps, scale bar: 200 μm. (**C**) Representative neurospheres formed after the first generation of neurospheres are passaged, scale bar: 200 μm. (**D**) The shape of CNSCs with adhered culture, scale bar: 100 μm. (**E**) The shape of CNSC-Ts with adhered culture, scale bar: 100 μm. (**F**) Upon reaching high density during adhered culture, CNSC-T started to aggregate into neurospheres, scale bar: 100 μm. (**G**) Representative neurospheres of CNSC-Ts with floating culture, scale bar: 200 μm. Representative images from at least three independent experiments were shown.

Throughout a mammal’s lifespan, NSCs exist in the subgranular zone (SGZ) of the dentate gyrus (DG) [26]. To generate the CNSCs, we prepared primary neural cells from the hippocampal tissues of adult Beagles (Figure 2A). NSCs gradually formed neurospheres with floating culture in the space between tissue scraps (Figure 2B) [27]. After 7 days of culturing, some of the larger neurospheres turned black due to a lack of nutrients. We then collected the supernatant containing the newly formed neurospheres for passaging, which exhibited a smooth and translucent morphology (Figure 2C). Passaging was determined based on neurosphere morphology and was performed when they showed signs of central necrosis or darkening cores. Following three passages of suspension culture, we transferred the floating CNSCs to laminin/poly-L-ornithine-coated dishes for adherent culture. As shown in Figure 2D, they exhibited a bipolar morphology with slender cell bodies.

### 3.3. Infection of CNSCs with SV40LT Virus

CNSCs were infected with an SV40LT retroviruses supernatant and then treated with puromycin to obtain cell lines stably expressing SV40LT (CNSC-T). The morphology of the CNSC-T cells (CNSC-Ts) was the same as that of the primary CNSCs (Figure 2E). When the cultured CNSC-Ts reached high density, the cells began to aggregate into neurospheres, a typical characteristic of NSCs (Figure 2F), and also formed neurospheres with floating culture (Figure 2G), demonstrating that they possessed the morphological characteristics and sphere-forming ability of CNSTs.

### 3.4. Identification of CNSCs and CNSC-Ts

**Figure 3:**
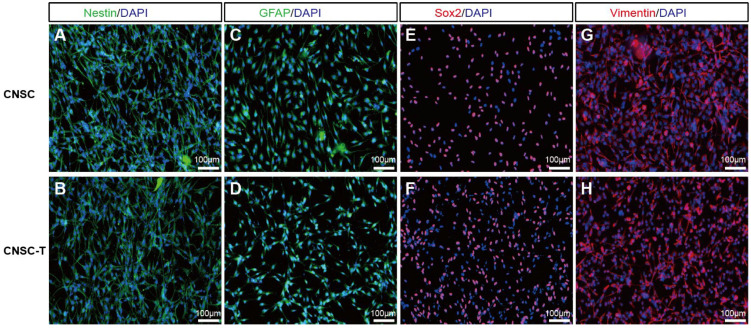
Immunocytochemical characterization of CNSCs and CNSC-Ts. Nestin, GFAP, Sox2, and Vimentin expression in CNSCs (**A**,**C**,**E**,**G**) and CNSC-Ts (**B**,**D**,**F**,**H**). The nuclei were counterstained with DAPI, scale bar: 100 μm. All experiments were performed from at least 3 independent biological replicates.

Neural stem cells express marker genes such as Nestin, Vimentin, Sox2, and GFAP [14,28,29]. Our immunofluorescence results revealed that both CNSCs and CNSC-Ts expressed Nestin, GFAP, Sox2, and Vimentin (Figure 3), with no significant difference between the two.

**Figure 4:**
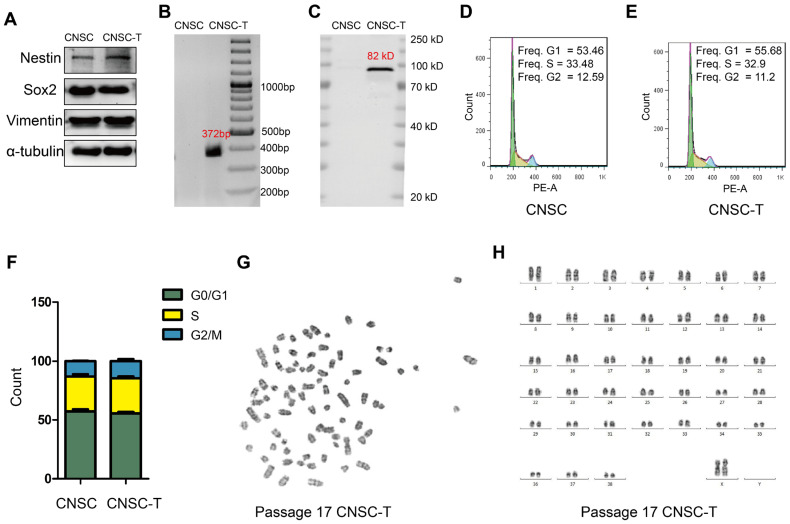
Identification of the CNSC-Ts. (**A**) Western blot was performed to detect the protein expression of Nestin, Vimentin, and Sox2, and α-tubulin was used as an internal control. (**B**) RT-PCR was performed to detect the RNA expression of SV40LT, and the length of RT-PCR production is 372 bp. (**C**) Western blot was performed to detect the protein expression of SV40LT, and the band size of SV40LT protein is 82 kD. (**D**) Flow cytometric analysis of CNSC cell cycle. (**E**) Flow cytometric analysis of CNSC-T cell cycle. (**F**) Histogram statistical analysis of the ratio of cells in the G0/G1, S, and G2/M phases of the cell cycle. (**G**) Karyotype analysis of CNSC-T cells at passage 17. (**H**) Mapping the CNSC-T chromosome (**G**) referred to the canine chromosome karyotype map, and there are 78 chromosomes in total. All experiments were performed from at least 3 independent biological replicates.

Western blotting also demonstrated that Nestin, Vimentin and Sox2 proteins were significantly expressed in both CNSCs and CNSC-Ts (Figure 4A). In order to verify whether SV40LT was expressed in the CNSC-Ts, RNAs of NSC-Ts and CNSCs were extracted and subjected to RT-PCR and Western blot analyses. This demonstrated that the CNSC-Ts expressed the SV40LT mRNA and protein (Figure 4B,C), implying that SV40LT had stably inserted into the CNSC-T genome. Cell cycle analysis of the CNSC-Ts at passage 5 and the CNSCs at passage 2, respectively, showed that there is no significant difference between the low-generation CNSC-Ts and low-generation CNSCs (Figure 4D,F). In addition, the CNSC-T clones at passage 17 displayed a normal karyotype (**2*n* = 78**, **XX**) (Figure 4G,H). Taken together, our results indicated that the CNSCs transformed by SV40LT displayed the morphology of NSCs, including a normal cell cycle and karyotype.

### 3.5. Determination of Proliferation Potential of CNSCs and CNSC-Ts

**Figure 5:**
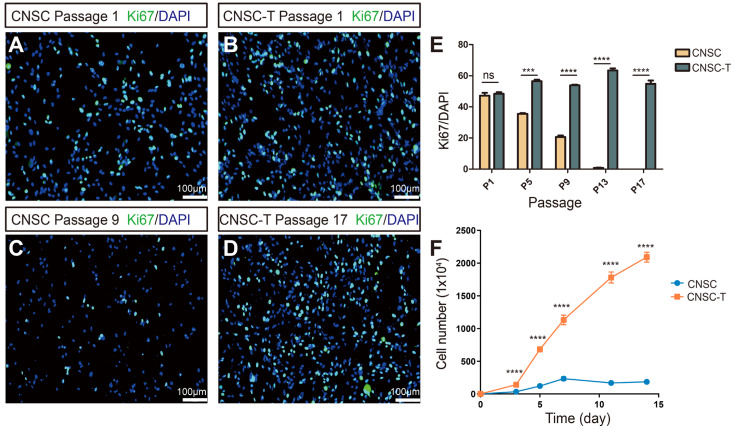
Detection of the proliferation potential of CNSC and CNSC-T. (**A**–**D**) Immunofluorescence analyses of Ki67^+^ CNSCs (**A**) and CNSC-Ts (**B**) at passage 1 (P1), and immunofluorescence analyses of Ki67^+^ CNSCs (**C**) at passage 9 (P9) and CNSC-Ts (**D**) at passage 17 (P17), respectively. The nuclei were counterstained with DAPI, scale bar: 100 μm. (**E**) Statistical analysis of the proportion of Ki67-positive cells at the P1, P5, P9, P13, and P17 CNSCs and CNSC-Ts respectively. (**F**) Growth curves of P5 CNSC and P7 CNSC-T cells within 14 days. All experiments were performed from 3 independent biological replicates (*n* = 3). Two-tailed Student’s *t*-test was employed to assess statistical significance. *p* < 0.05 was considered to be significant. *** *p* < 0.001 and **** *p* < 0.0001; ns, no significant difference.

Ki67 is a marker of cell proliferation [8,17]; for different cellular generations, the ratios of Ki67-positive ratio (Ki67^+^) cells in CNSCs and CNSC-Ts were different. At passage 1, there was no significant difference in the proportion of Ki67^+^ CNSCs and Ki67^+^ CNSC-Ts, and the proportion of proliferating cells was about 45–50% (Figure 5A,B,E). As the passage number increased, the Ki67^+^ CNSC-Ts maintained a stable level (Figure 5D,E), while the proportion of Ki67^+^ CNSCs gradually decreased with the increase in passages (Figure 5C,E). At passage 5, the proportion of Ki67^+^ CNSCs was about 37%, while at passage 13, they had nearly entered a state of growth arrest (Figure 5E). Growth curve results indicated that the CNSC-Ts continued to proliferate for at least 14 days, but the number of CNSCs gradually decreased (Figure 5F). These data revealed that primary CNSCs can only be cultured in vitro for a few generations; as the number of passages increased, CNSCs gradually died. However, the proportion of proliferating CNSC-Ts remained stable in vitro, indicating that the limited proliferative lifespan of CNSCs was overcome by immortalization.

### 3.6. Differentiation Potential of CNSCs and CNSC-Ts

**Figure 6:**
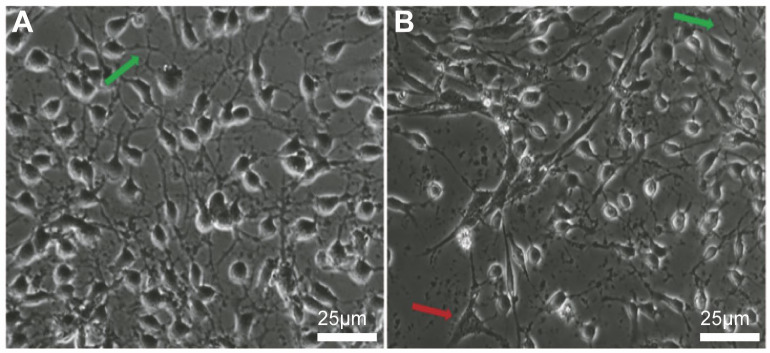
The differentiated morphology of CNSC-Ts. (**A**,**B**) The morphology of CNSC-Ts after differentiation. The green arrows point to obvious neuronal neurites, and the red arrow point to obvious glial cell morphology. Scale bar: 25 μm.

**Figure 7:**
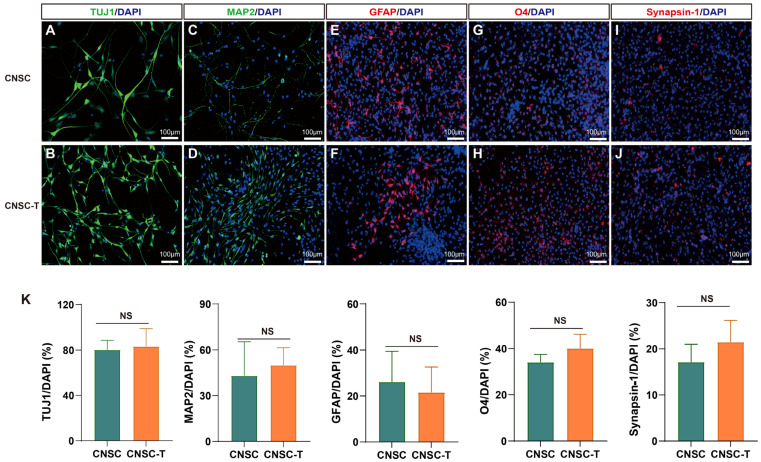
CNSCs at passage 6 (P6) and CNSC-Ts at passages 18 (for TUJ1, MAP2, GFAP, and O4 staining) and 23 (for Synapsin-1 staining) were used to assess differentiation potential. The CNSCs (**A**,**C**,**E**,**G**,**I**) and CNSC-Ts (**B**,**D**,**F**,**H**,**J**) can undergo differentiation into neurons, astrocytes, and oligodendrocytes under differentiation condition for 11 days as shown by immunofluorescence staining of TUJ1 (β-Tubulin III), MAP2, GFAP, O4, and Synapsin-1. The nuclei were counterstained with DAPI, scale bar: 100 μm. All experiments were performed from at least 3 independent biological replicates. (**K**) Statistical analysis of the proportions of TUJ1-, MAP2-, GFAP-, O4- and Synapsin-1-positive cells in CNSCs and CNSC-Ts, respectively. Two-tailed Student’s *t*-test was employed to assess statistical significance. *p* < 0.05 was considered to be significant. NS, no significant difference.

An important property of NSCs is their multipotentiality, whereby their progeny of NSCs are able to undergo terminal differentiation into neurons, astrocytes, and oligodendrocytes [18]. After 11 days of differentiation, CNSC-Ts showed obviously differentiated neuronal morphology, some with obvious neurites, and some cells showing glial cell morphology (Figure 6A,B). Immunofluorescence results demonstrated that both the differentiated CNSCs and CNSC-Ts expressed the neuronal marker genes β-Tubulin III (TUJ1) and MAP2, the astrocyte marker gene GFAP, the oligodendrocyte marker gene O4, and the synaptic marker gene Synapsin-1 (Figure 7A–J). Our statistical analysis of the proportions of TUJ1-, MAP2-, GFAP-, O4-, and Synapsin-1-positive cells showed no significant difference between CNSCs and CNSC-Ts (Figure 7K). These results provided evidence that CNSCs and CNSC-Ts have the multipotentiality to undergo differentiation into neurons, astrocytes, and oligodendrocytes.

## 4. Discussion

Dogs have coexisted with humans for thousands of years, and this unique relationship has driven remarkable behavioral and cognitive adaptations [30]. The domestication of canines involved selective pressures that favored reduced aggression and enhanced social-cognitive abilities compared to their wolf ancestors [31]. These behavioral changes are thought to be accompanied by neuroanatomical adaptations [32]. The canine brain is therefore an intriguing model for studying neural plasticity and neurogenesis in the context of evolved cognitive capabilities. Canine models offer several advantages over rodent models, particularly in terms of the genetics and physiology of the central nervous system [33]. For instance, the brains of dogs share more anatomical and physiological similarities with human brains than those of rodents [34]. Additionally, dogs cohabit with humans and are exposed to similar environmental factors, such as diet, pollutants, and lifestyle influences, and this shared environment is impossible to replicate in laboratory rodent studies and adds a critical dimension to translational research [35]. Notably, cognitive dysfunction in aging dogs naturally recapitulates key features of human Alzheimer’s disease, including amyloid-β accumulation, cognitive decline, and neuroinflammation, without the need for artificial transgene expression [36]. These features make the canine nervous system a valuable model for understanding the complex structure and pathology of the human nervous system. In particular, NSCs hold promise for both stem cell therapies and basic research in fields such as neural development [13,37]. However, the proliferative potential of hippocampal NSCs is limited in vitro.

In this study, we successfully established an immortalized hippocampal neural stem cell line through the in vitro isolation, screening, and culture of canine hippocampal neural stem cells, followed by subsequent infection with SV40 large T antigen (SV40LT) retroviruses. Immunocytochemical analysis showed that canine hippocampal neural stem cells (CNSCs) and immortalized canine hippocampal neural stem cells (CNSC-Ts) were intensely stained with NSC, neuronal, and oligodendrocyte markers. The growth rate of low-passage CNSC-Ts was similar to that of low-passage CNSCs, while high-passage CNSC-T clones exhibited a normal karyotype. Importantly, CNSC-Ts were shown to be pluripotent and capable of indefinite expansion in vitro, whereas primary CNSCs could only be passaged 8–10 times before gradually ceasing proliferation and eventually dying. The CNSC-T lines we established will be applicable in both various basic research studies and stem cell therapies.

## 5. Conclusions

We established an immortalized canine neural stem cell line by isolating hippocampal neural stem cells from 5-month-old Beagles and transducing them with a retrovirus expressing the SV40 large T antigen. The cell line maintained its stemness and differentiation potential while exhibiting unlimited proliferative capacity, thereby overcoming the limited lifespan of primary cultures.

## Data Availability

The original contributions presented in this study are included in the article. Further inquiries can be directed to the corresponding author.
